# Ultrassom Point-of-Care na Cardiologia: Quando o Posicionamento Não Reflete o Estado da Arte

**DOI:** 10.36660/abc.20260312

**Published:** 2026-08-31

**Authors:** Guilherme Pinheiro Machado, Guilherme Heiden Telo, Gustavo Neves de Araujo, Fernando Luis Scolari, Anderson Donelli da Silveira, Rodrigo Wainstein, Marco Wainstein

**Affiliations:** 1 Hospital de Clínicas de Porto Alegre Porto Alegre RS Brasil Hospital de Clínicas de Porto Alegre, Porto Alegre, RS – Brasil; 2 Hospital da Unimed Grande Florianópolis São José SC Brasil Hospital da Unimed Grande Florianópolis, São José, SC – Brasil; 3 Universidade Federal do Rio Grande do Sul Porto Alegre RS Brasil Universidade Federal do Rio Grande do Sul, Porto Alegre, RS – Brasil

**Keywords:** Infarto do Miocárdio com Supradesnível do Segmento ST, Diagnóstico por Ultrassom, Ecocardiografia, Cardiopatias

**Prezado Editor,**

Lemos com interesse a recente publicação do Posicionamento sobre o Uso do Ultrassom "Point of Care" (POCUS) na Medicina Cardiovascular – 2026,^1^ nos Arquivos Brasileiros de Cardiologia. Trata-se de uma iniciativa relevante, que reconhece o papel crescente do POCUS como ferramenta fundamental na prática cardiovascular contemporânea. Entretanto, apesar de seus méritos, consideramos que o posicionamento apresenta uma limitação importante: a falta de discussão e de incorporação de um conjunto expressivo de evidências relacionadas à avaliação de pacientes cardiológicos em diferentes cenários clínicos, particularmente no infarto agudo do miocárdio com supradesnivelamento do segmento ST (IAMCSST).

Nos últimos anos, diversos estudos demonstraram que abordagens baseadas em POCUS podem aprimorar substancialmente a estratificação inicial desses pacientes. A classificação LUCK,^2^ que integra a ultrassonografia pulmonar à classificação de Killip, demonstrou capacidade de reclassificar o risco de mortalidade com desempenho superior ao da avaliação clínica isolada e foi recentemente incorporada às diretrizes norte-americanas de síndrome coronariana aguda,^3^ evidenciando sua relevância internacional. Adicionalmente, Scolari et al.^4^ demonstraram que a congestão pulmonar avaliada por ecografia pulmonar se associa tanto aos estágios de choque definidos pela classificação SCAI quanto à mortalidade intra-hospitalar no IAMCSST, sendo capaz de refinar a estratificação prognóstica mesmo entre pacientes inicialmente classificados como de baixo risco.

De forma complementar, a classificação LUV,^5^ que combina a avaliação da congestão pulmonar com a estimativa do débito cardíaco por meio da integral velocidade-tempo da via de saída do ventrículo esquerdo, apresentou elevado desempenho prognóstico, superando diversos modelos de avaliação baseados em POCUS para a predição de mortalidade intra-hospitalar e desenvolvimento de choque cardiogênico no contexto do IAMCSST.^6^ Evidência mais recente publicada por Araujo et al.^7^ ampliou a compreensão da fisiopatologia do infarto de ventrículo direito ao demonstrar, por meio da ecografia pulmonar, que a concepção clássica de "pulmões limpos" nesse cenário frequentemente não corresponde à realidade clínica. Além disso, o protocolo FASTEMI^8^ propõe uma abordagem estruturada e sistemática de avaliação cardiotorácica à beira do leito em pacientes com IAMCSST, permitindo a identificação precoce de complicações mecânicas e a caracterização de perfis hemodinâmicos de maior risco. Da mesma forma, uma revisão recente da literatura^9^ que consolida o papel do POCUS como ferramenta integradora na avaliação cardiovascular não foi citada no documento.

Outra limitação relevante refere-se à não inclusão da maior coorte já publicada avaliando o escore Venous Excess Ultrasound no contexto do IAMCSST.^10^ Vale destacar que esse estudo foi publicado nos próprios Arquivos Brasileiros de Cardiologia, tornando sua omissão particularmente relevante sob a perspectiva científica e editorial. Além disso, investigações recentes têm ampliado o escopo de aplicação do POCUS para além do cenário coronariano agudo, incluindo sua utilização na avaliação pré-procedimento de intervenções estruturais, como o implante transcateter de válvula aórtica, reforçando sua versatilidade e potencial impacto clínico.^11,12^ Nesse contexto, a não incorporação dessas evidências não parece representar apenas uma limitação pontual, mas uma lacuna relevante em relação ao estado atual do conhecimento disponível. Considerando que parte dessas contribuições foi publicada na própria revista da Sociedade Brasileira de Cardiologia, tal ausência adquire ainda maior relevância.

Em conclusão, entendemos que a não inclusão de evidências relevantes, já disponíveis e amplamente reconhecidas pela literatura, reduz a abrangência do documento e reforça a necessidade de futuras atualizações. O Brasil tem desempenhado papel de destaque na produção científica relacionada ao POCUS aplicado à cardiologia, e a adequada incorporação dessas contribuições em documentos oficiais é fundamental para alinhar recomendações clínicas ao estado da arte e, potencialmente, contribuir para a melhoria dos desfechos dos pacientes.

## References

[1] 1 Alcantara ML, Bernardo MPL, Beck ALS, Santos SN, Morhy SS, Lira EB Filho, et al. Positioning Statement on the Use of Point-of-Care Ultrasound in Cardiovascular Medicine – 2026. Arq Bras Cardiol. 2026;123(4):e20260222. doi: 10.36660/abc.20260222. 10.36660/abc.20260222 PMC13671151 42307308

[2] 2 Araujo GN, Silveira AD, Scolari FL, Custodio JL, Marques FP, Beltrame R, et al. Admission Bedside Lung Ultrasound Reclassifies Mortality Prediction in Patients with ST-Segment-Elevation Myocardial Infarction. Circ Cardiovasc Imaging. 2020;13(6):e010269. doi: 10.1161/CIRCIMAGING.119.010269. 10.1161/CIRCIMAGING.119.010269 32536197

[3] 3 Rao SV, O’Donoghue ML, Ruel M, Rab T, Tamis-Holland JE, Alexander JH, et al. 2025 ACC/AHA/ACEP/NAEMSP/SCAI Guideline for the Management of Patients with Acute Coronary Syndromes: A Report of the American College of Cardiology/American Heart Association Joint Committee on Clinical Practice Guidelines. J Am Coll Cardiol. 2025;85(22):2135-237. doi: 10.1016/j.jacc.2024.11.009. 10.1016/j.jacc.2024.11.009 40013746

[4] 4 Scolari FL, Machado GP, Pagnoncelli A, Chies A, Araujo GN, Silveira AD, et al. Lung Ultrasound Evaluation of SCAI Shock Stages Predicts Mortality in ST-Segment Elevation Myocardial Infarction. JACC Cardiovasc Imaging. 2023;16(2):260-2. doi: 10.1016/j.jcmg.2022.09.005. 10.1016/j.jcmg.2022.09.005 36648045

[5] 5 Machado GP, Telo GH, Araujo GN, Barbato JPR, Amon A, Martins A, et al. A Combination of Left Ventricular Outflow Tract Velocity Time Integral and Lung Ultrasound to Predict Mortality in ST Elevation Myocardial Infarction. Intern Emerg Med. 2024;19(8):2167-76. doi: 10.1007/s11739-024-03719-z. 10.1007/s11739-024-03719-z 39044051

[6] 6 Machado GP, Telo GH, Araujo GN, Silveira AD, Wainstein M. Comparing Ultrasound-Based Prognostic Classifications in STEMI: Is LUV Better than LUCK? Intern Emerg Med. 2025;20(5):1645-7. doi: 10.1007/s11739-025-03920-8. 10.1007/s11739-025-03920-8 40131637

[7] 7 Araujo GN, Telo GH, Machado GP, Silveira AD, Scolari FL, Nassif MP, et al. Right Ventricular ST-Segment Elevation Myocardial Infarction and Pulmonary Congestion: Insights from a Prospective Cohort of Patients Evaluated by Admission Lung Ultrasound. J Am Soc Echocardiogr. 2026;39(4):422-4. doi: 10.1016/j.echo.2025.12.006. 10.1016/j.echo.2025.12.006 41456686

[8] 8 Amon AB, Machado GP, Telo GH, Saadi MP, Azevedo WTA, Martins A, et al. Clinical Impact of a Cardiothoracic Ultrasound Protocol in Patients with ST-Segment Elevation Myocardial Infarction: The Focused Assessment in STEMI (FASTEMI) Protocol. Intern Emerg Med. 2025. doi: 10.1007/s11739-025-04212-x. 10.1007/s11739-025-04212-x 41291168

[9] 9 Wainstein MV, Machado GP, Telo GH, Silveira AD, Nasi LA, Araujo GN. Point-of-Care Ultrasound in the Evaluation of STEMI Patients. Front Cardiovasc Med. 2025;12:1627396. doi: 10.3389/fcvm.2025.1627396. 10.3389/fcvm.2025.1627396 PMC12436283 40964524

[10] 10 Machado GP, Telo GH, Barbato JPDR, Saadi MP, Araujo GN, Chies A, et al. Venous Excess Ultrasound (VExUS) Score in Patients with ST-Elevation Myocardial Infarction to Predict in-Hospital Mortality. Arq Bras Cardiol. 2025;122(6):e20250093. doi: 10.36660/abc.20250093. 10.36660/abc.20250093 PMC12269907 40736126

[11] 11 Machado GP, Crivelaro PCF, Araujo GN, Telo GH, Bergoli LCC, Saadi MP, et al. Prognostic Role of Pre-Procedural Lung Ultrasound in Patients Undergoing Transcatheter Aortic Valve Implantation. Front Cardiovasc Med. 2025;12:1626497. doi: 10.3389/fcvm.2025.1626497. 10.3389/fcvm.2025.1626497 PMC12339517 40799638

[12] 12 Lillo R, Cangemi S, Graziani F, Locorotondo G, Pedicino D, Aurigemma C, et al. Pulmonary Congestion Assessed by Lung Ultrasound in Patients with Severe Aortic Stenosis Undergoing Transcatheter Aortic Valve Implantation: Prevalence and Prognostic Implications. Eur J Heart Fail. 2024;26(10):2107-17. doi: 10.1002/ejhf.3365. 10.1002/ejhf.3365 39014551

