# Supplementary material for: Ultrassom Point-of-Care na Cardiologia: Quando o Posicionamento Não Reflete o Estado da Arte
Source: Arq Bras Cardiol. 2026 Aug 31;123(8):e20260312. [Article in Portuguese] doi: 10.36660/abc.20260312 (PMC13668379; doi:10.36660/abc.20260312)
Supplement: Translation

# Translation: Point-of-Care Ultrasound in Cardiology: When a Positioning Statement Does Not Reflect the State of the Art

**DOI:** 10.36660/abc.20260312i

**Authors:** Guilherme Pinheiro Machado, Guilherme Heiden Telo, Gustavo Neves de Araujo, Fernando Luis Scolari, Anderson Donelli da Silveira, Rodrigo Wainstein, Marco Wainstein

**Affiliations:** 1 Porto Alegre RS Brazil Hospital de Clínicas de Porto Alegre, Porto Alegre, RS – Brazil; 2 São José SC Brazil Hospital da Unimed Grande Florianópolis, São José, SC – Brazil; 3 Porto Alegre RS Brazil Universidade Federal do Rio Grande do Sul, Porto Alegre, RS – Brazil

**Keywords:** ST Elevation Myocardial Infarction, Diagnostic Ultrasound, Echocardiography, Heart Diseases

**Dear Editor,**

We read with interest the recently published Positioning Statement on the Use of Point-of-Care Ultrasound in Cardiovascular Medicine – 2026,^1^ in Arquivos Brasileiros de Cardiologia. This is a relevant initiative that acknowledges the growing role of point-of-care ultrasound (POCUS) as a fundamental tool in contemporary cardiovascular practice. Nevertheless, despite its merits, we believe that the document presents an important limitation: the lack of discussion and incorporation of a substantial body of evidence regarding the assessment of cardiac patients across different clinical scenarios, particularly ST-segment elevation myocardial infarction (STEMI).

Over recent years, several studies have demonstrated that POCUS-based approaches can substantially improve the initial risk stratification of these patients. The LUCK classification,^2^ which integrates lung ultrasound with the Killip classification, has been shown to reclassify mortality risk with superior performance compared with clinical assessment alone and has recently been incorporated into North American acute coronary syndrome guidelines,^3^ highlighting its international relevance. Additionally, Scolari et al.^4^ demonstrated that pulmonary congestion assessed by lung ultrasound is associated with both shock stages defined by the SCAI classification and in-hospital mortality in STEMI, being capable of refining prognostic stratification even among patients initially classified as low risk.

Complementarily, the LUV classification,^5^ which combines the assessment of pulmonary congestion with cardiac output estimation through the left ventricular outflow tract velocity-time integral, demonstrated high prognostic performance, outperforming several POCUS-based assessment models for predicting in-hospital mortality and the development of cardiogenic shock in the setting of STEMI.^6^ More recent evidence published by Araujo et al.^7^ expanded the understanding of right ventricular infarction pathophysiology by demonstrating, through lung ultrasound, that the classical concept of "clean lungs" in this scenario often does not correspond to clinical reality. Furthermore, the FASTEMI protocol^8^ proposes a structured and systematic bedside cardiothoracic assessment approach for patients with STEMI, enabling the early identification of mechanical complications and the characterization of higher-risk hemodynamic profiles. Likewise, a recent literature review^9^ consolidating the role of POCUS as an integrative tool in cardiovascular assessment was not cited in the document.

Another relevant limitation is the omission of the largest cohort ever published evaluating the Venous Excess Ultrasound score in the context of STEMI.^10^ Notably, such a study was published in Arquivos Brasileiros de Cardiologia itself, which makes its omission particularly significant from both scientific and editorial perspectives. In addition, recent investigations have expanded the scope of POCUS applications beyond the acute coronary setting, including its use in the pre-procedural assessment of structural interventions such as transcatheter aortic valve implantation, further reinforcing its versatility and potential clinical impact.^11,12^ In this context, the failure to incorporate these data appears not merely as an isolated limitation, but rather as a meaningful gap relative to the current state of available knowledge. Considering that some of these contributions were published in the official journal of the Brazilian Society of Cardiology, their absence becomes even more significant.

In conclusion, we believe that the omission of relevant evidence, already available and widely recognized in the literature, reduces the comprehensiveness of the document and underscores the need for future updates. Brazil has played a prominent role in the scientific production related to POCUS applied to cardiology, and the appropriate incorporation of these contributions into official documents is essential to align clinical recommendations with the state of the art and, potentially, contribute to improved patient outcomes.
