# Supplementary material for: Ultrassom Point-of-Care na Cardiologia: Quando o Posicionamento Não Reflete o Estado da Arte
Source: Arq Bras Cardiol. 2026 Aug 31;123(8):e20260312. [Article in Portuguese] doi: 10.36660/abc.20260312 (PMC13668379; doi:10.36660/abc.20260312)
Supplement: Related texts

# Reply: Carta-resposta

**Authors:** Monica Luiza de Alcantara

**Affiliations:** 1 Quinta D’Or Hospital Rio de Janeiro RJ Brasil Quinta D’Or Hospital, Rio de Janeiro, RJ – Brasil; 2 Americas Medical Services Rio de Janeiro RJ Brasil Americas Medical Services, Rio de Janeiro, RJ – Brasil; Rede D’Or 3 Rio de Janeiro RJ Brasil Rede D’Or, Rio de Janeiro, RJ – Brasil

**Prezado Editor,**

Agradecemos o interesse e a leitura atenta de nossa publicação Posicionamento sobre o Uso do Ultrassom "Point of Care" (POCUS) na Medicina Cardiovascular – 2026,^1^ bem como as pertinentes considerações apresentadas pelos autores da correspondência. Recebemos com satisfação contribuições que ampliem o debate científico acerca do papel do POCUS na prática cardiovascular contemporânea.

Inicialmente, gostaríamos de reconhecer a relevância da produção científica brasileira mencionada pelos autores, particularmente no contexto do infarto agudo do miocárdio com supradesnivelamento do segmento ST (IAMCSST), da congestão pulmonar, da caracterização de perfis hemodinâmicos e das aplicações prognósticas do ultrassom à beira do leito. Trata-se de contribuições de elevado mérito científico, com reconhecimento nacional e internacional, incluindo estudos recentemente incorporados a documentos de referência. Concordamos que a cardiologia brasileira tem assumido protagonismo crescente na produção de conhecimento relacionado ao POCUS cardiovascular e consideramos extremamente positivo que essas evidências continuem a ser difundidas e incorporadas a futuras recomendações e documentos científicos.

Entretanto, é importante esclarecer que o objetivo do Posicionamento^1^ não foi realizar uma revisão sistemática ou exaustiva da literatura disponível sobre POCUS em cardiologia, tampouco abordar de forma aprofundada cenários clínicos específicos. Concebido por múltiplos departamentos da Sociedade Brasileira de Cardiologia (SBC), o documento teve caráter predominantemente educacional, com foco na padronização do ensino, na aquisição e interpretação de imagens e na integração clínica do método à prática cardiovascular. Dessa forma, priorizou-se a construção de um modelo de treinamento aplicável a diferentes níveis de competência, em vez do detalhamento de protocolos voltados a contextos clínicos específicos.

Nesse sentido, algumas áreas de rápida expansão científica receberam abordagem necessariamente mais sintética, em consonância com o escopo previamente estabelecido. Em particular, no cenário do IAMCSST, optou-se por não aprofundar classificações prognósticas avançadas e protocolos integrados direcionados a essa condição. Embora reconheçamos a relevância e a robustez das classificações LUCK e LUV, bem como as contribuições relacionadas ao protocolo FASTEMI, ao escore Venous Excess Ultrasound e à aplicação da ecografia pulmonar no choque cardiogênico, entendemos que essas ferramentas representam estratégias de refinamento prognóstico que extrapolam os objetivos de um documento transversal voltado à formação e à capacitação em POCUS.

Em relação às publicações mais recentes mencionadas pelos autores, cabe destacar que o conteúdo do manuscrito foi consolidado ao longo de 2024, com base nas evidências disponíveis à época. Sua elaboração ocorreu por meio de um amplo processo institucional e multidisciplinar, envolvendo diferentes departamentos da SBC. A complexidade inerente à construção consensual do documento, somada às etapas de revisão técnica e normativa conduzidas pelo Conselho de Normatizações e Diretrizes da SBC, resultou em um processo editorial necessariamente extenso e rigoroso, o que limitou a incorporação de parte das evidências publicadas posteriormente.

Ainda assim, procuramos disponibilizar um documento essencialmente prático e aplicável à realidade assistencial. O Posicionamento foi estruturado a partir dos princípios de aquisição, interpretação e implementação do método em cenários críticos, incluindo choque, parada cardiorrespiratória, dispneia e outras emergências cardiovasculares. Adicionalmente, o posicionamento é complementado por amplo material multimídia, incluindo 68 vídeos educacionais destinados a facilitar a curva de aprendizado de profissionais não especialistas em métodos de imagem. Acreditamos que essa abordagem fornece uma base sólida e padronizada para a futura incorporação de protocolos mais especializados e de novos avanços científicos.

Por fim, agradecemos novamente aos autores pela oportunidade de uma discussão acadêmica respeitosa e construtiva. Entendemos que o debate científico qualificado e plural é fundamental para o amadurecimento do conhecimento na área e para o fortalecimento contínuo da produção cardiovascular brasileira.

## References

[1] 1 Alcantara ML, Bernardo MPL, Beck ALS, Santos SN, Morhy SS, Lira EB Filho, et al. Positioning Statement on the Use of Point-of-Care Ultrasound in Cardiovascular Medicine – 2026. Arq Bras Cardiol. 2026;123(4):e20260222. doi: 10.36660/abc.20260222.10.36660/abc.20260222PMC1367115142307308

# Reply

**Authors:** Monica Luiza de Alcantara

**Affiliations:** 1 Rio de Janeiro RJ Brazil Quinta D’Or Hospital, Rio de Janeiro, RJ – Brazil; 2 Rio de Janeiro RJ Brazil Americas Medical Services, Rio de Janeiro, RJ – Brazil; 3 Rio de Janeiro RJ Brazil Rede D’Or, Rio de Janeiro, RJ – Brazil

**Dear Editor,**

We thank the authors of the correspondence for their interest in and careful reading of our publication, Positioning Statement on the Use of Point-of-Care Ultrasound in Cardiovascular Medicine – 2026,^1^ as well as for the pertinent considerations they have raised. We welcome contributions that broaden the scientific discussion regarding the role of point-of-care ultrasound (POCUS) in contemporary cardiovascular practice.

First, we would like to acknowledge the relevance of the Brazilian scientific production highlighted by the authors, particularly in the context of ST-segment elevation myocardial infarction (STEMI), pulmonary congestion, hemodynamic profile characterization, and the prognostic applications of bedside ultrasound. These are contributions of substantial scientific merit, with both national and international recognition, including studies that have recently been incorporated into reference documents. We agree that Brazilian cardiology has assumed an increasingly prominent role in the generation of knowledge related to cardiovascular POCUS, and we consider it highly valuable that such evidence continues to be disseminated and incorporated into future recommendations and scientific documents.

However, it is important to clarify that the objective of the Positioning Statement^1^ was not to provide a systematic or exhaustive review of the available literature on POCUS in cardiology, nor to address specific clinical scenarios in depth. Developed through the collaboration of multiple departments of the Brazilian Society of Cardiology (SBC), the document was conceived with a predominantly educational purpose, focusing on the standardization of training, image acquisition and interpretation, and the clinical integration of the method into cardiovascular practice. Accordingly, priority was given to the development of a training framework applicable across different levels of competency rather than to the detailed discussion of protocols tailored to specific clinical contexts.

In this regard, some rapidly expanding areas of scientific knowledge necessarily received a more concise treatment, consistent with the predefined scope of the document. Specifically, in the setting of STEMI, we chose not to elaborate on advanced prognostic classifications and integrated protocols designed for this condition. Although we recognize the relevance and robustness of the LUCK and LUV classifications, as well as the contributions related to the FASTEMI protocol, the Venous Excess Ultrasound score, and the application of lung ultrasound in cardiogenic shock, we understand that such tools represent prognostic refinement strategies that extend beyond the objectives of a cross-sectional document focused on education and training in POCUS.

Regarding the more recent publications mentioned by the authors, please note that the content of the manuscript was consolidated throughout 2024, based on the evidence available at that time. Its development involved a broad institutional and multidisciplinary process encompassing different SBC departments. The inherent complexity of building consensus within such a document, combined with the technical and regulatory review stages conducted by the SBC Council on Standards and Guidelines, resulted in a necessarily lengthy and rigorous editorial process. This limited the incorporation of some evidence published subsequently.

Nevertheless, we sought to provide a document that is fundamentally practical and applicable to real-world clinical care. The Positioning Statement was structured around the principles of image acquisition, interpretation, and implementation of the method in critical scenarios, including shock, cardiac arrest, dyspnea, and other cardiovascular emergencies. In addition, the Positioning Statement is complemented by extensive multimedia material, including 68 educational videos designed to facilitate the learning curve of health professionals who are not specialists in cardiovascular imaging. We believe that this approach provides a solid and standardized foundation for the future incorporation of more specialized protocols and emerging scientific advances.

Finally, we would like to thank the authors once again for the opportunity to engage in a respectful and constructive academic discussion. We believe that a qualified, pluralistic scientific debate is essential for advancing knowledge in the field and strengthening Brazilian cardiovascular research.
